# [^18^F]Fluorodeoxyglucose Positron Emission Tomography for Diagnosis and Monitoring of Acute *Staphylococcus aureus* Vascular Graft Infection in a Rat Model

**DOI:** 10.1093/infdis/jiaf594

**Published:** 2025-11-26

**Authors:** Emma Faddy, Mikkel Illemann Johansen, Christoffer Gadeberg, Rikke Louise Meyer, Lars Østergaard, Cecilie Bay-Richter, Louise Kruse Jensen, Mikkel Holm Vendelbo, Nis Pedersen Jørgensen

**Affiliations:** Department of Clinical Medicine, Infectious Diseases, Aarhus University, Aarhus N, Denmark; Department of Infectious Diseases, Aarhus University Hospital, Aarhus N, Denmark; Department of Orthopedic Surgery, Regional Hospital of Randers, Randers NØ, Denmark; Department of Infectious Diseases, Aarhus University Hospital, Aarhus N, Denmark; Department of Clinical Medicine, Infectious Diseases, Aarhus University, Aarhus N, Denmark; Department of Infectious Diseases, Aarhus University Hospital, Aarhus N, Denmark; Interdisciplinary Nanoscience Center (INANO), Aarhus University, Aarhus C, Denmark; Department of Biology, Aarhus University, Aarhus C, Denmark; Department of Clinical Medicine, Infectious Diseases, Aarhus University, Aarhus N, Denmark; Department of Infectious Diseases, Aarhus University Hospital, Aarhus N, Denmark; Translational Neuropsychiatry Unit, Department of Clinical Medicine, Aarhus University, Aarhus N, Denmark; Department of Veterinary and Animal Sciences, University of Copenhagen, Frederiksberg C, Denmark; Department of Nuclear Medicine and PET Centre, Aarhus University Hospital, Aarhus N, Denmark; Department of Biomedicine, Aarhus University, Aarhus C, Denmark; Department of Infectious Diseases, Aarhus University Hospital, Aarhus N, Denmark; Department of Orthopedic Surgery, Aarhus University Hospital, Aarhus N, Denmark

**Keywords:** positron-emission tomography, staphylococcus aureus, staphylococcus epidermidis, prosthesis-related infections, blood vessel prosthesis

## Abstract

**Objectives:**

Vascular graft or endograft infections (VGEIs) pose a detrimental complication when using vascular grafts and are challenging to diagnose and treat. This study examined the progression of infection and the antimicrobial response in VGEI using [^18^F]Fluorodeoxyglucose (chemical name, 2-Deoxy-2-[18F]fluoroglucose) (FDG) positron emission tomography (PET), ex vivo bacterial quantification, and histology in a VGEI rat model.

**Methods:**

In this experimental study, 97 male Sprague–Dawley rats had a polytetrafluorethylene graft surgically implanted in the carotid artery. The graft was either preinoculated with *Staphylococcus aureus*, *S. epidermidis*, or saline. Up to 31 days after surgery, rats were FDG-PET-scanned. Subsequently, they were killed, and the implants were retrieved for analysis. A subgroup of infected rats received daptomycin and rifampicin from days 20 to 29.

**Results:**

Tracer uptake around the implant, measured by maximum standardized uptake value (SUV_max_), declined over time in all groups. Between groups, SUV_max_ was highest in untreated *S. aureus*-infected rats. When comparing antibiotic-treated and uninfected rats by day 31, there was no difference in SUV_max_, although the treated rats were still infected. Histology revealed widespread inflammation by day 10 in *S. aureus*-infected rats, which decreased by days 20 and 31 with encapsulation of the infection, alongside increased plasma interleukin-10.

**Conclusions:**

FDG-PET differentiated untreated *S. aureus*-infected rats from uninfected ones but failed to monitor infection progression, as SUV_max_ declined over time despite a constant bacterial load. FDG-PET could not distinguish between uninfected rats and those with suppressed infection, likely due to reduced inflammation and encapsulation of the infection.

Vascular graft or endograft infections (VGEIs) represent a catastrophic complication to the use of vascular grafts and are associated with high mortality and morbidity [1–5]. The risk of infection ranges between 1% and 6%, with a mortality rate up to 75% [1–5]. Some of the most common pathogens in VGEI are *Staphylococcus aureus* and *S. epidermidis* [2, 6].

VGEI treatment is complex; the gold standard involves surgical removal, graft replacement, and extended antibiotic treatment [7–9]. Therefore, prompt and accurate diagnosis is imperative.

The European Society for Vascular Surgery recommends using the MAGIC criteria to diagnose VGEI [10]. One criterion is suspicious metabolic activity in positron emission tomography/computed tomography (PET/CT), utilizing a radioactively labelled glucose analogue [^18^F]Fluorodeoxyglucose (chemical name, 2-Deoxy-2-[18F]fluoroglucose) (FDG).

Sunde et al. found that the focal FDG uptake pattern around the graft and the maximum standardized uptake value (SUV_max_) were reliable parameters in diagnosing VGEI with PET/CT [11]. However, some uptake is also seen in noninfected vascular grafts, potentially affecting the diagnostic accuracy of FDG-PET/CT in diagnosing VGEI [12].

PET/CT has also been suggested for monitoring treatment response and infection status.

Husmann et al. [13] found that PET/CT was helpful in deciding whether to continue or escalate antimicrobial therapy based on continued focal FDG uptake despite a reduction in CRP. However, PET/CT was less applicable when deciding to stop antibiotic therapy, as focal FDG uptake remained in 50% of patients who had antimicrobial therapy discontinued anyway [13]. A crucial shortcoming in enhancing the performance of nuclear imaging on VGEI is the lack of clinically relevant animal models, which are essential for understanding disease progression under controlled conditions. This study followed and described the course of infection and response to antimicrobial therapy in a VGEI rat model using FDG-PET.

## METHODS

### Ethics

The Danish Animal Experiments Inspectorate approved this study under permission 2021-15-0201-00894. It was carried out following the guidelines of Aarhus University, under the supervision of faculty veterinarians (Department of Biomedicine, Aarhus University), and following the Animal Research: Reporting of In Vivo Experiments (ARRIVE) guidelines.

### Animals

A total of 97 adult male Sprague–Dawley rats (Janvier Labs, France) were used. They weighed 200–300 g upon arrival, with a 1-week acclimatization before surgery. The rats were housed at the animal facilities of the Department of Biomedicine, Aarhus University, in double-decker cages at standard room temperature and humidity, a 12-hour day/night cycle, and free access to food and water.

### Bacterial Strain, Growth Media, and Antibiotics

The strains included clinical methicillin-susceptible *S. aureus* (MSSA) isolate SAU060112 [14] and clinical *S. epidermidis* isolate MEN42. MSSA was cultivated on tryptic soy agar and grown in tryptic soy broth (TSB, T8907, Sigma Aldrich). *S. epidermidis* was cultivated on Brain Heart Infusion (BHI, 53286, Millipore) agar and grown in BHI. Antibiotics were diluted in 0.9% saline (Natriumklorid, B. Braun Medical). The antibiotics were daptomycin (“Cubicin”, Merck Sharp & Dohme, Copenhagen, Denmark), rifampicin (“Rifadin”, Sanofi S.r.I., Milan, Italy), and a single dose of preoperatively administered cefuroxime (“Cefuroxim”, Fresenius Kabi, Copenhagen, Denmark).

### Inoculation of Vascular Grafts

Vascular grafts made of polytetrafluoroethylene (PTFE) were preinoculated following a previously described protocol [15], only changing the isolate and culture medium.

### Rat Vascular Graft Infection Model

The rat vascular graft infection model, including the surgical procedures, has been previously described [15]. Briefly, the preinoculated vascular graft was surgically inserted into the right carotid artery, with return of blood flow through the implant.

**Figure 1.**
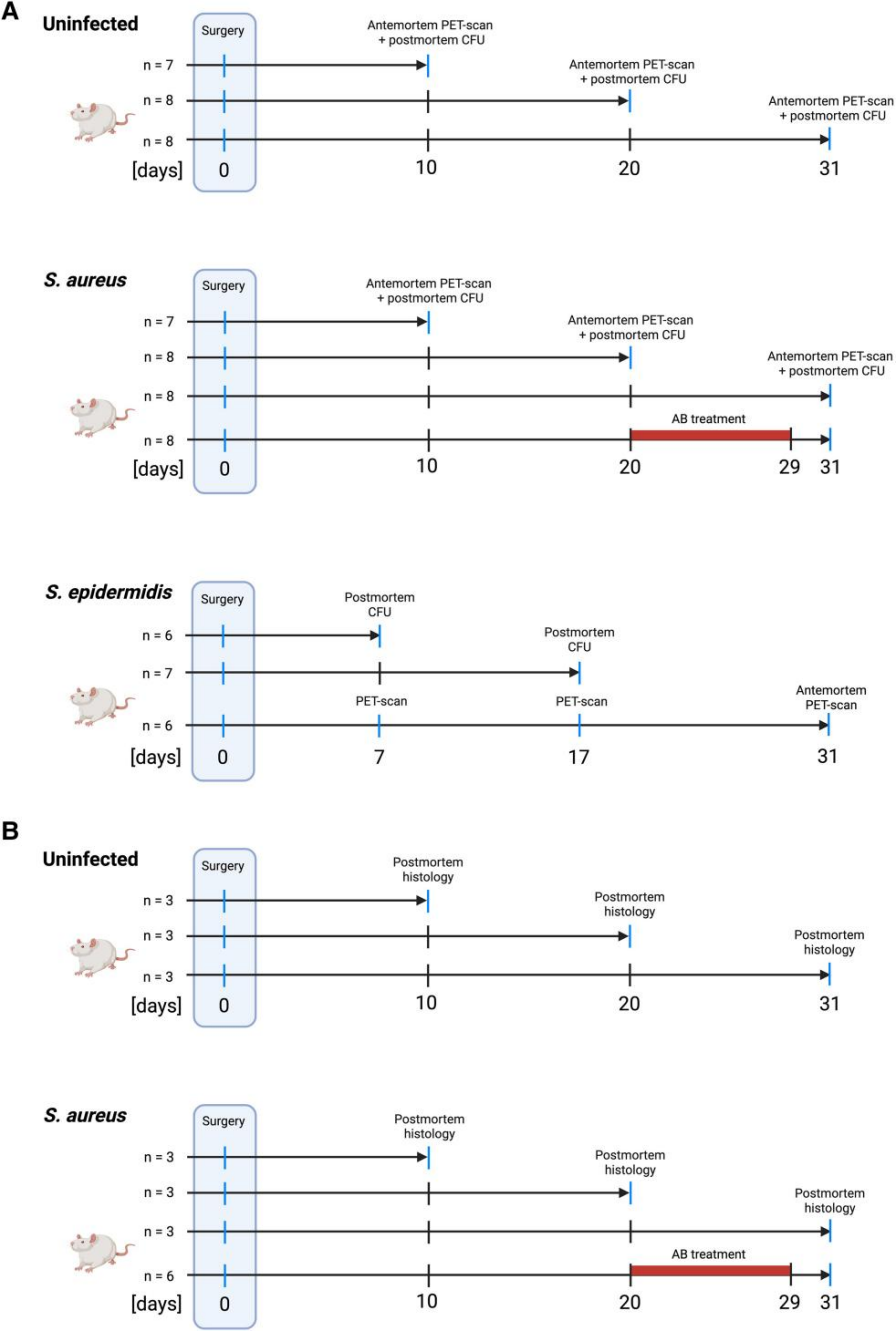
Study design, overview of animal experiments. (*A*) Animals used for PET and bacterial quantification. (*B*) Animals used for histology. A detailed list of reasons for animal exclusion from the study is found in Supplementary Figure 1. Created in BioRender. Faddy, E. (2025) https://BioRender.com/bjto6ra.

The study outline can be seen in Figure 1*A* and *B*. Animals were used either for antemortem PET scan and postmortem bacterial quantification (Figure 1*A*), or postmortem histological analysis of vascular graft and perigraft tissue (Figure 1*B*). For animals undergoing PET scan and postmortem bacterial quantification, a total of n = 23 uninfected, n = 31 *S. aureus*-infected, and n = 19 *S. epidermidis*-infected rats were planned. After 10 (n = 7 of each), 20 (n = 8 of each), or 31 days (n = 8/16), uninfected and *S. aureus*-infected rats were PET-scanned (Figure 1*A*). After the scan, vascular grafts were surgically removed, blood samples were acquired by intracardial aspiration, and the animals were killed by cervical dislocation. The vascular grafts were immediately stored at −80°C.

A subgroup of PET-scanned *S. aureus*-infected rats (n = 8) received daptomycin (100 mg/kg/24 h) subcutaneously and rifampicin (25 mg/kg/12 h) intraperitoneally for 10 days (days 20–29 postinfection) [15].

For *S. epidermidis*, n = 6 rats were transported to the PET facilities by day 7 and scanned consecutively on days 7, 17, and 31 (Figure 1*A*). After the day 31 scan, samples were collected, and the rats were killed, as previously described. Colony-forming units (CFU) data for days 7 (n = 6) and 17 (n = 7) were obtained from a separate group of rats infected with *S. epidermidis*. Due to hygiene regulations, serial scans could only be conducted on *S. epidermidis*-infected rats.

In rats used for postmortem histology, a total of n = 9 were uninfected and n = 15 were infected with *S. aureus* (Figure 1*B*). By days 10, 20, or 31, these rats were killed, and the left and right arteries, including the implant and soft tissue, were removed, fixated, and hematoxylin and eosin stained as described previously [16]. N = 6 *S. aureus*-infected rats used for histology were treated with antibiotics as described previously.

Criteria for exclusion and inclusion are described in detail in Supplementary Figure 1. Details on animal welfare and weighing are described in the Supplementary Text and Supplementary Figure 2.

### PET Methods

Animals were anesthetized with isoflurane and injected intravenously with approximately 20 MBq FDG. After a 1-hour distribution period under anesthesia, rats were scanned for 20 minutes using microPET/Magnetic Resonance Imaging (microPET/MRI, Mediso Medical Imaging Systems, Budapest, Hungary).

The PET images were reconstructed with a 3-dimensional ordered subset expectation algorithm (Tera-Tomo 3D; Mediso Medical Imaging Systems) with 4 iterations, 6 subsets, and a voxel size of 0.6 × 0.6 × 0.6 mm. Data were corrected for dead time, decay, and randoms using a delayed coincidence window without corrections for attenuation and scatter.

Hermes software (Hermes Medical Solutions, Stockholm, Sweden) was used for analysis. Visual inspection defined a volume of interest (VOI) containing the implant and surrounding tissue. The localization was determined in relation to the rat's right humerus. SUV_max_ was defined from each VOI as a measure of the maximum FDG-tracer uptake normalized to the injected activity and weight.

### Quantification of Bacterial Load

Bacterial load was quantified as previously described [15]. Vascular grafts were thawed, placed in 1 ml PBS, vortexed, sonicated, serially diluted, and plated onto 5% horse blood agar (SSI Diagnostica, Copenhagen, Denmark). Grafts and excess sonicate were added to 20 ml TSB media and incubated at 180 rpm and 37°C for 24 hours to increase the detection limit to 1 CFU/implant.

### Histology

Perivascular neutrophil infiltration, macrophage infiltration, and fibroplasia were semiquantitatively scored as absent, mild, moderate, or massive. Delta Pix Insight software (Smørum, Denmark) measured the largest perpendicular distance from the graft-tissue interface until no perivascular inflammatory changes (cellular infiltration or fibrosis) were observed. A pathologist blinded to group allocation and study design performed all registrations.

### Immunohistochemistry Against *S. aureus*

The immunohistochemistry (IHC) protocol, published by Jensen et al. [17], was followed as described previously [16]. A pathologist blinded to group allocation and study design performed all IHC evaluations.

### Cytokine Analysis

Blood samples were collected in ethylenediaminetetraacetic (EDTA) tubes and centrifuged for 15 minutes at 2500 G twice at 20°C, and the supernatant was collected in between. Plasma samples were stored immediately at −80°C until further analysis.

Cytokines tumor necrosis factor (TNF)-α, interferon (IFN)-γ, interleukin (IL)-6, and IL-10 were measured using standard ELISA kits from Invitrogen (Massachusetts, USA), following the manufacturer's instructions. Briefly, a 25 μl sample was diluted 1:5 in sample diluent. All samples and standards were analyzed in duplicate and read spectrophotometrically at 450 nm.

### Data Management

Statistical analysis and graphs were created using GraphPad Prism Version 10.1.1.

Data were analyzed for normality by inspecting a QQ plot.

Unpaired *t*-test was used to compare values at time points with only 2 groups if normally distributed. If not, Mann-Whitney test was applied.

For comparison of 3 or more groups, one-way ANOVA with post hoc Tukey's test was used for normally distributed data. Kruskal-Wallis test with post hoc Dunn's test was used for data not normally distributed. *P*-values below 0.05 were considered statistically significant.

Power calculations for rats needed to detect a difference in SUV_max_ between infected and uninfected were based on pilot data from animals scanned by day 10. In the pilot, *S. aureus*-infected rats had a mean (±SD) SUV_max_ of 4.4 (±1.2). Uninfected rats had a mean SUV_max_ of 1.5. With an alpha of 0.05 and power of 80%, the total sample per group needed was 3. Under the assumption that differences in SUV_max_ would decrease over time, the samples required were doubled as a minimum.

## RESULTS

### SUV_max_ Peaked on Day 10 and Subsequently Decreased in All Groups. By Day 31, SUV_max_ was Comparable Between Uninfected and Antibiotic-Treated Rats

**Figure 2.**
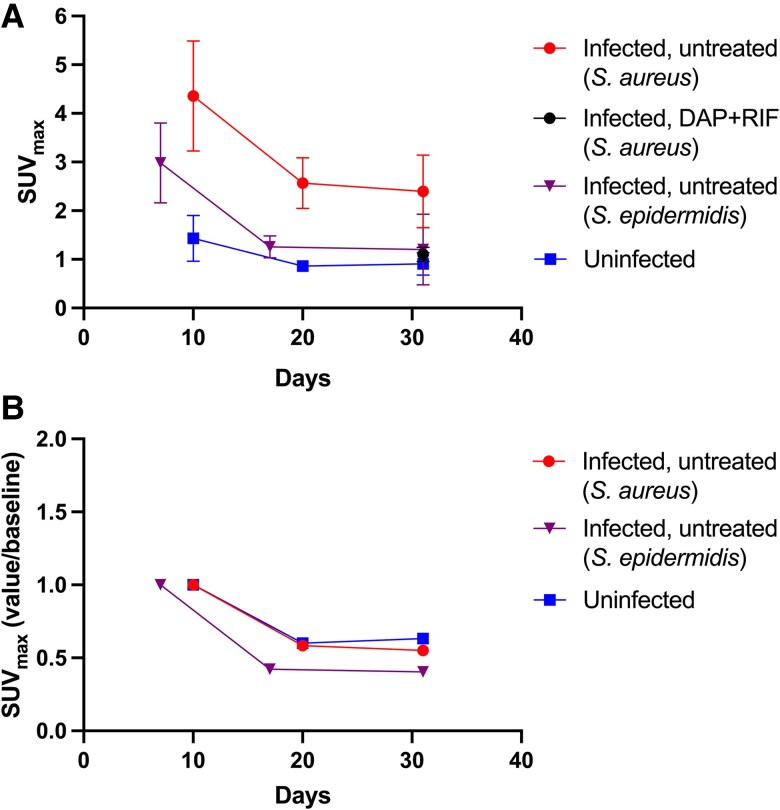
FDG uptake around the implant. (*A*) Each point represents mean SUV_max_ of each group scanned at the defined time points. Bars represent SD. *S. aureus*, day 10 (n = 6). *S. aureus*, day 20 (n = 5). *S. aureus*, day 31 (n = 6). *S. aureus*, DAP + RIF (n = 8). *S. epidermidis*, day 7 (n = 6). *S. epidermidis*, day 17 (n = 5). *S. epidermidis*, day 31 (n = 4). Uninfected, day 10 (n = 6). Uninfected, day 20 (n = 6). Uninfected, day 31 (n = 8). (*B*) Fractional SUV_max_. Each point represents the mean fraction of SUV_max_ at defined time points compared to baseline (days 7/10).

**Figure 3.**
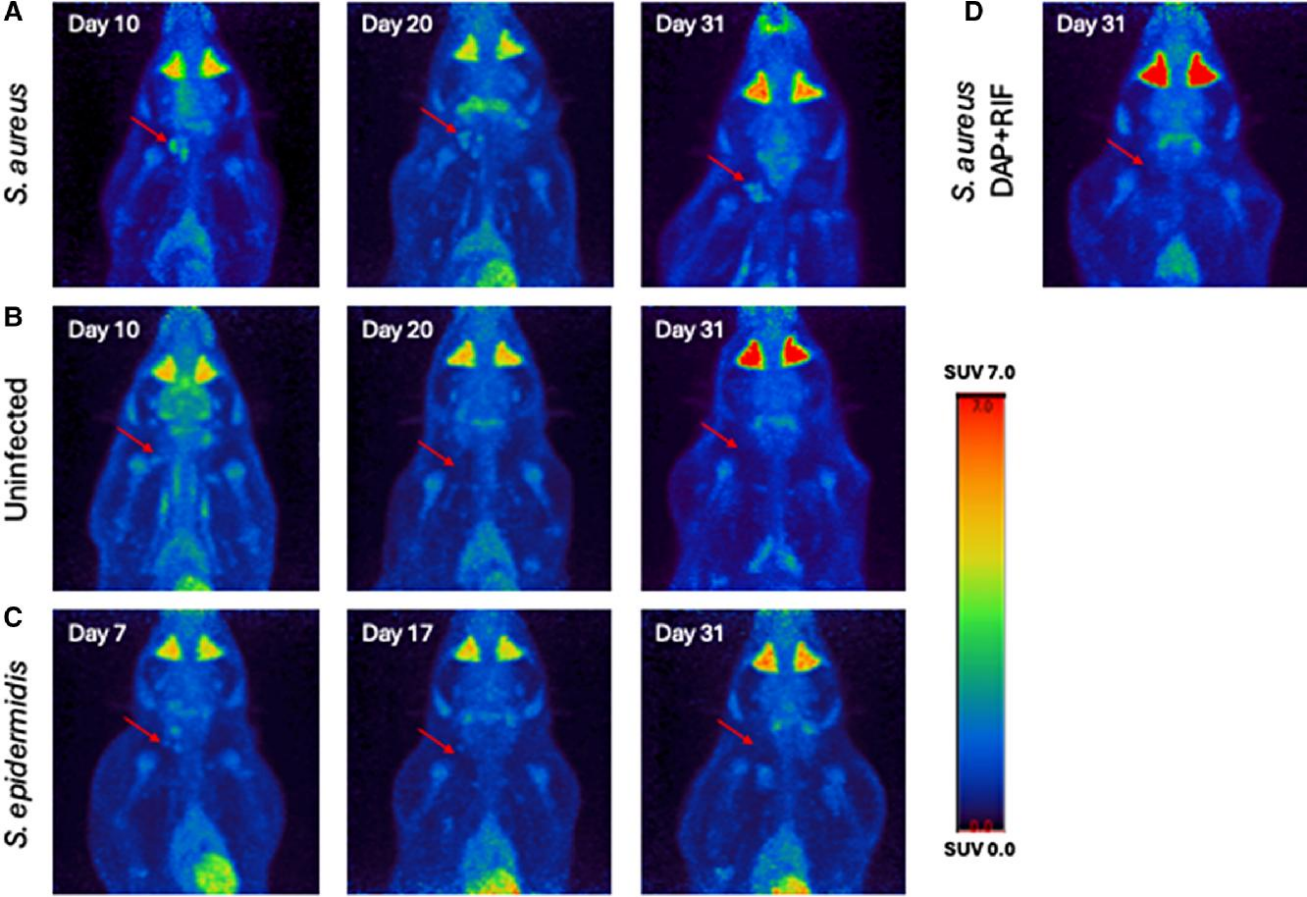
Images from PET scan of individual rats from each group. The arrow indicates the location of the implant. (*A*) *S. aureus*-infected rats at days 10, 20, and 31. (*B*) Uninfected rats at days 10, 20, and 31. (*C*) *S. epidermidis*-infected rat at days 7, 17, and 31. Images from *S. epidermidis*-infected rat are from the same rat at different time points. (*D*) *S. aureus*-infected rat treated with daptomycin and rifampicin on day 31.

At day 10, SUV_max_ was higher in *S. aureus*-infected rats than in uninfected ones (mean ± SD: 4.357 ± 1.132 vs. 1.433 ± 0.473, *P* < .001) (Figures 2*A* and 3). By day 20, SUV_max_ decreased in both groups, but the difference remained significant (2.568 ± 0.521, *S. aureus* vs. 0.862 ± 0.114, uninfected, *P* < .001). This trend continued on day 31, with SUV_max_ stabilizing in both groups (2.398 ± 0.746, *S. aureus* vs. 0.905 ± 0.232, uninfected, *P* < .001).

In *S. epidermidis*-infected rats, mean SUV_max_ was 2.980 ± 0.824 by day 7, declining to 1.258 ± 0.226 by day 17, and 1.203 ± 0.725 by day 31 (Figure 2*A*).

Rats infected with *S. aureus* but treated with daptomycin and rifampicin had a mean SUV_max_ of 1.100 ± 0.149 at day 31, comparable to that of uninfected rats and *S. epidermidis*-infected rats (*P* = .37). The fractional reduction in SUV_max_ in untreated *S. aureus*-infected rats and uninfected rats was similar (Figure 2*B*).

### Untreated, *S. aureus*-Infected Rats Had a Stable Infection, While Antibiotic Treatment Reduced Bacterial Load

**Figure 4.**
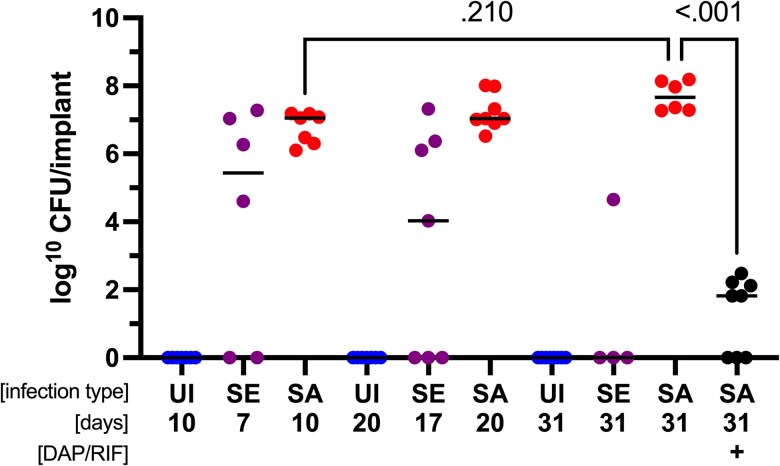
Colony-forming units (CFU) for each group. Data points represent mean CFU of 3 technical replicates from 1 implant. Bars represent median. Kruskal-Wallis test with post hoc Dunn's test. Uninfected, day 10 (n = 7). *S. epidermidis*, day 7 (n = 6). *S. aureus*, day 10 (n = 7). Uninfected, day 20 (n = 7). *S. epidermidis*, day 17 (n = 7). *S. aureus*, day 20 (n = 8). Uninfected, day 31 (n = 8). *S. epidermidis*, day 31 (n = 4). *S. aureus*, day 31 (n = 6). *S. aureus* DAP + RIF, day 31 (n = 8). DAP/RIF = daptomycin + rifampicin; SA = *S. aureus*; SE = *S. epidermidis*; UI = uninfected.

Uninfected rats’ implants remained sterile with no bacterial growth (Figure 4). *S. aureus*-infected rats had a median (range) log CFU/implant of 7.1 (6.1–7.2) at day 10, 7.0 (6.5–8.0) at day 20, and 7.7 (7.3–8.2) by day 31, with no significant difference from days 10 to 31 (*P* = .21). Treatment with daptomycin and rifampicin led to a median log CFU/implant of 1.8 (0.0–2.5) at day 31. Despite this reduction, 5 of 8 rats remained infected. The CFU difference between untreated and treated *S. aureus*-infected rats at day 31 was significant (*P* < .001).

### *S. epidermidis*-Infected Rats Presented with an Unstable Infection

By day 7, *S. epidermidis*-infected rats had a median (range) log CFU/implant of 5.4 (0.0–7.3) (Figure 4). It decreased to 4.0 (0.0–7.3) and 0.0 (0.0–4.7) by days 17 and 31, respectively. Several implants were sterile at all time points in rats inoculated with *S. epidermidis*. By day 7, 2 out of 6 implants (33.3%) were sterile. By day 17, 3 out of 7 implants (42.9%) were sterile; by day 31, this was the case for 3 out of 4 implants (75%) (*P* = .58, Fisher's exact test).

### Trend Towards Higher SUV_max_ in Rats with Suppressed Infection Compared to Cured Rats

For the antibiotic-treated rats by day 31, the infections could be divided into 2 categories: cured (no bacteria present on the implant) and well-suppressed (bacteria present on the implant). Mean ± SD SUV_max_ in rats with a well-suppressed infection was 1.18 ± 0.10, compared to 0.97 ± 0.14 in cured rats (Supplementary Figure 3). Despite a trend toward higher SUV_max_ in still-infected rats, this difference was insignificant (*P* = .052).

### Early Widespread Inflammation with Massive Neutrophil and Macrophage Infiltration in Implants and Surrounding Tissue in Untreated *S. aureus*-Infected Rats Compared to Those Uninfected

**Figure 5.**
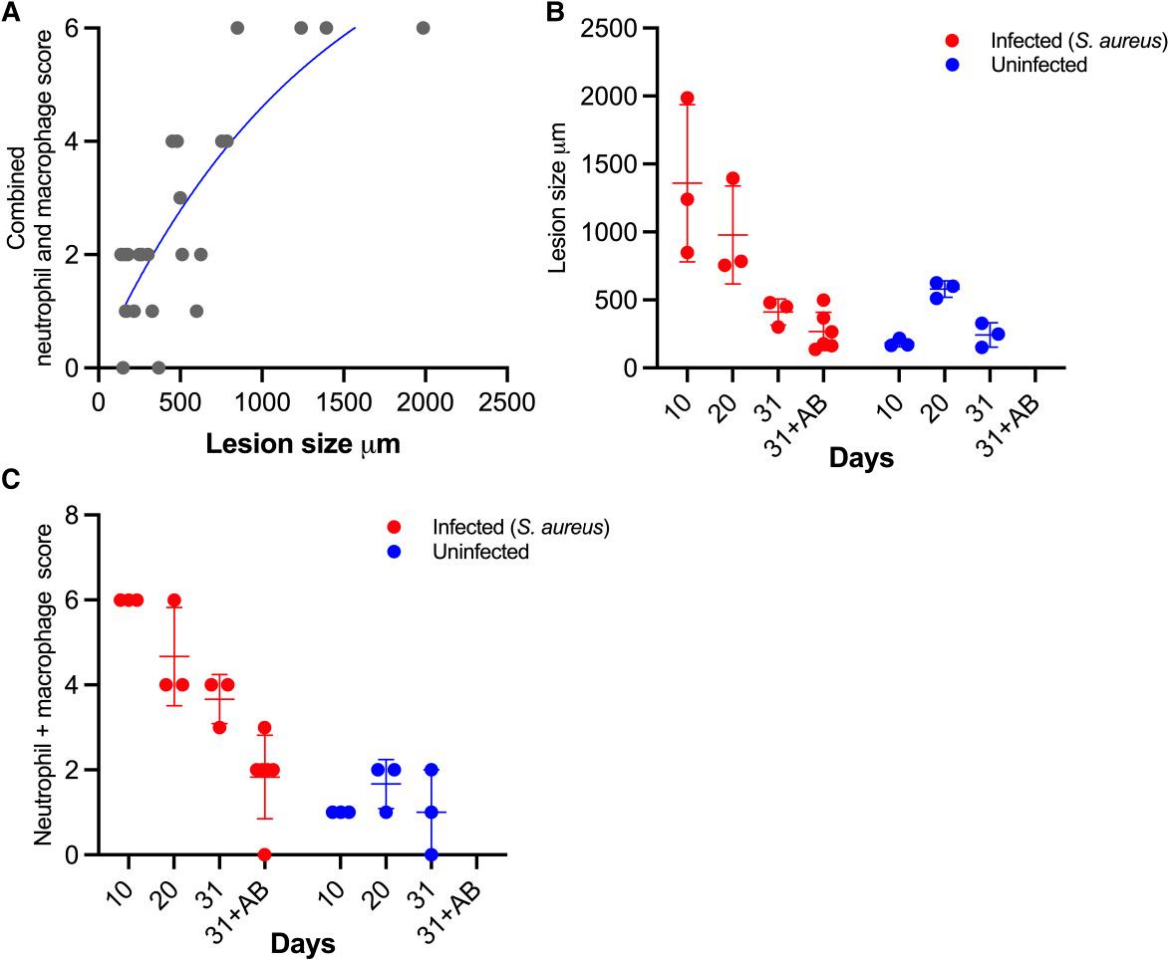
Histological analysis. Each point represents data from 1 rat. Bars represent mean and SD. (*A*) Neutrophil and macrophage infiltration score (max 3 for each) vs. lesion size. (*B*) Lesion size (inflammatory distribution). (*C*) Combined neutrophil and macrophage score (max score = 6).

In uninfected rats, 1 rat on day 10 and all rats on days 20 and 31 had broken elastic laminas of tunica media, and all rats on days 20 and 31 had almost complete or complete loss of the elastic bands. The tissue surrounding the implants was a mix of tunica adventitia and periadventitial tissue. Neutrophil and macrophage infiltration were absent or minor for all time points (Figure 5*C*). The width of the inflammatory distribution outwards from the implant-tissue interface was relatively constant, ranging from 165 to 627 µm (Figure 5*B*).

**Figure 6.**
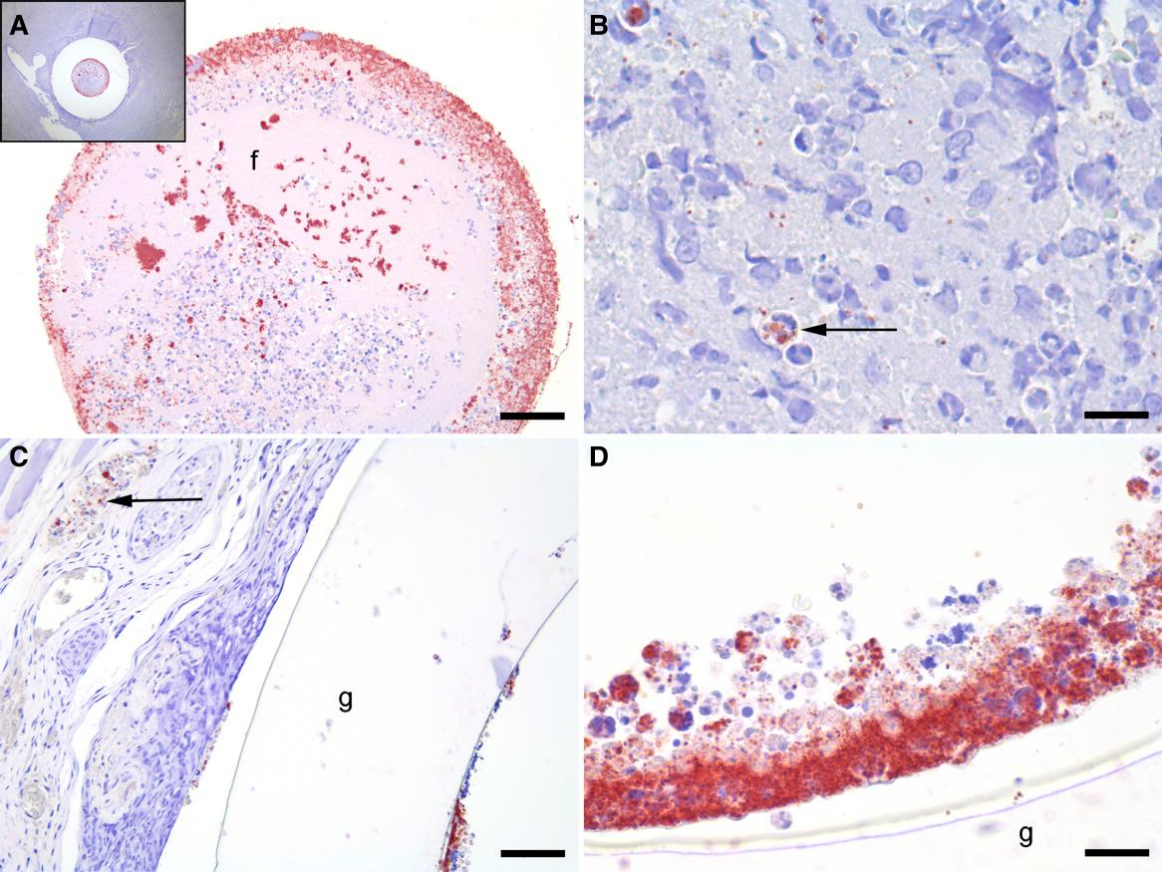
Immunohistochemistry based on antibodies towards *S. aureus*. (*A*) and (*B*) represents an animal with *S. aureus*-infected vascular graft for 10 days, while (*C*) and (*D*) represents an animal with infected vascular graft and systemic antibiotic treatment at 31 days. (*A*) The insert illustrates the graft lumen and the surrounding inflammatory cell infiltration. Fibrin (f), inflammatory cells, erythrocytes, and numerous bacterial colonies (red), primarily located towards the graft's inner surface, fill the lumen. Bar = 150 µm. (*B*) Close-up of the inflammatory cell infiltration and debris seen next to the graft in the insert of (*A*). Red *S. aureus* bacteria are visible intracellularly (arrow) and as single bacteria. Bar = 30 µm. (*C*) Minimal inflammation around the graft (g). Red bacteria are located in the graft lumen, at the tissue-graft interface, and in adjacent vessels (arrow). Bar = 200 µm. (*D*) Close-up of the bacteria inside the graft (g) lumen reveals a large bacterial aggregate along numerous intracellular bacteria. Bar = 30 µm.

For untreated *S. aureus*-infected rats, normal tunica media architecture was lost in all rats, ie, broken and lost elastic bands. Therefore, the tissue surrounding the implants was a mix of tunica adventitia and periadventitial tissue. The fibrotic response increased from moderate in rats on day 10 to massive in most rats on days 20 and 31. Neutrophil and macrophage infiltration was massive in all rats at day 10, indicated by high cell density over a large area (Figure 6*A* and *B*). At day 20, 1 rat had a neutrophil and macrophage infiltration comparable to rats on day 10, whereas the remaining rats at day 20 were like those on day 31 with moderate infiltration and lower cell density (Figure 5*C*). The inflammatory distribution outwards from the implant-tissue interface was greater at day 10 compared to day 31, which was more comparable to uninfected rats (Figure 5*B*). The inflammatory distribution width of rats at day 20 was in-between rats at days 10 and 31; however, 1 rat at day 20 was comparable to day 10 (the one with massive leucocyte infiltration).

### Despite Low Inflammatory Response in Antibiotic-Treated Rats, Bacteria Were Still Present in and Surrounding the Implant in Most Treated Animals

By day 31, vessel patho-morphology in antibiotic-treated rats resembled that of uninfected rats. Neutrophil and macrophage infiltrations and fibroplasia were minor overall; however, 1 rat exhibited moderate neutrophil infiltration. The mean width of inflammatory distribution from the implant-tissue interface was also comparable to that of uninfected rats on day 31 (Figure 5*B*).

Using IHC, all untreated *S. aureus*-infected rats were consistently positive for *S. aureus.* The bacteria were located inside the implant lumen and perivascularly, both as colonies and intracellularly within neutrophils and macrophages (Figure 6*A* and *B*). In the group receiving antibiotic administration, 4 out of 6 rats were positive for *S. aureus*, with the bacteria located as previously described for nontreated animals (Figure 6*C* and *D*).

### Increased Plasma Interleukin-10 in Rats Infected with *S. aureus* by Day 31

**Figure 7.**
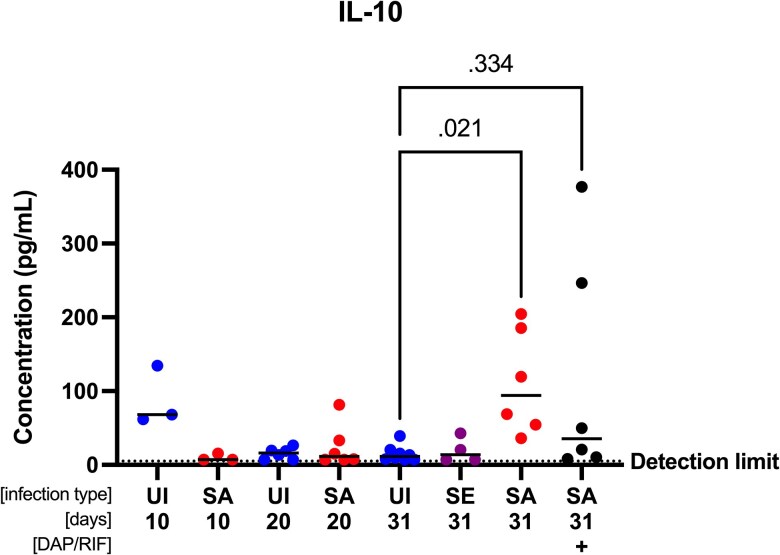
Interleukin-10 (IL-10) concentrations in plasma. Each point shows mean value of 1–2 technical replicates from 1 rat. Bars represent median. The lower limit of detection was 7.278 pg/ml. Plasma samples below the measurable concentration were set at a default 7.278 pg/ml to avoid skewing of data. Kruskal-Wallis with post hoc Dunn's test of day 31 groups. DAP/RIF = daptomycin + rifampicin; SA = *S. aureus*; SE = *S. epidermidis*; UI = uninfected.

Plasma interleukin-10 (IL-10) levels were low in the first 20 days postsurgery, except in uninfected rats, where levels peaked at 68.14 pg/ml by day 10 (Figure 7). In untreated *S. aureus*-infected rats, IL-10 concentrations were significantly higher by day 31 compared to uninfected rats (*P* = .021). At this point, antibiotic-treated infected rats also had trending higher IL-10 levels, median 35.37 pg/ml, with a notable variation in range (8.3–376.8 pg/ml, *P* = .33). IL-6, IFN-γ, and TNF-α levels were below detection levels for all samples.

## DISCUSSION

To our knowledge, this study is the first to explore the use of FDG-PET to monitor the course of infection in a true intravascular VGEI rat model, using both *S. aureus* and *S. epidermidis*.

FDG-PET reliably identified rats with untreated *S. aureus* VGEI within 31 days. Despite a gradual increase in bacterial burden, SUV_max_ fell throughout the infection. In antibiotic-treated rats, SUV_max_ decreased to levels observed in uninfected rats, even with persistent, although significantly reduced, infection. Histological analysis indicated that changes in SUV_max_ correlated with decreased inflammatory responses in both treated and untreated rats, supported by an increase in IL-10.

This study explores the use of nuclear imaging in diagnosing and monitoring VGEI. While several studies assess the diagnostic performance of FDG-PET/CT for VGEI [18], data on infection monitoring are scarce. EANM guidelines recommend caution when interpreting scans of patients receiving antibiotics [19]. Given the heterogeneity of clinical data in this context, our study highlights the limitations of FDG-PET in detecting a well-suppressed infection.

A previous preclinical murine study of *S. aureus* VGEI found FDG-PET/MRI useful for diagnosing VGEI. However, scans were only performed on day 10 postinfection, and the animals were either sterile or infected but untreated [20].

A clinical study found it safe to discontinue antibiotic treatment in 44 out of 68 VGEI patients following a complete response on a follow-up PET/CT scan. In contrast to our study, these patients had undergone longer antibiotic therapy of 340 days (210–435, median [IQR]) when the PET/CT scan was performed [13]. Further studies are needed to establish the application of FDG-PET imaging for monitoring treatment response for VGEI.

The histological analysis revealed several findings. A suppurative inflammatory reaction was present at all time points for *S. aureus*-infected rats. However, the local distribution of the inflammatory response differed with time. At day 10, the inflammatory distribution into the periadventitial tissue was extremely high compared to 2 rats on day 20 and all on day 31. The reduced distribution on days 20 and 31 was due to 2 factors: (1) more fibrosis is seen as an attempt to encapsulate the infection and (2) a smaller area of massive neutrophil and macrophage infiltration. The infection was still present on days 20 and 31 but remarkably more contained and enclosed than on day 10. The measurements of IL-10, an anti-inflammatory mediator, support this observation. It has previously been shown that IL-10 is upregulated during *S. aureus* infection due to its immune evasive capabilities [21, 22]. In this study, we saw an increase in plasma concentrations of IL-10 in *S. aureus*-infected rats by day 31 compared to uninfected rats. This could contribute to the simultaneous decrease in inflammation observed in the histological analysis, in line with the previously described immunomodulatory behavior observed in *S. aureus*.

The study has certain limitations. Biofilm infections often become chronic. However, this study only examined VGEI during the first 31 days of infection. While exploring a prolonged infection period is of relevance, this study provides valuable insights into the context of an acute VGEI.

Here, PTFE was used as graft material. Dacron is another standard vascular graft material that warrants investigation, as FDG uptake patterns can vary between the 2 types [23]. This includes a more inhomogeneous and diffuse FDG uptake in Dacron grafts than in PTFE grafts [23].

We examined only a few individual cytokines (IL-10, IL-6, TNF-α, and IFN-γ), of which only IL-10 yielded measurable results. The inflammatory response is a complex process that involves interactions between numerous cytokines. In future studies, we recommend investigating this response further with a broader array of cytokine panels. In addition, immunohistochemical analyses of pro- and anti-inflammatory cytokines in periprosthetic tissue could provide further insight into the local inflammatory response and its influence on FDG uptake.

PET scans were performed without anatomical coregistration. The original protocol included combined PET/MRI scans for the precise location of the implant; however, this was not feasible as the rats exceeded the MRI scanner's weight limit of 350 g by day 20. Nonetheless, the VOI, which included the vascular implant and periprosthetic tissue, was precisely identified using PET alone, as its position was clearly related to the rat's right humerus, which was visible on PET.

We faced challenges in establishing a stable *S. epidermidis* infection, as many rats cleared it spontaneously, likely due to the low virulence of the bacteria and the rats’ strong immune response, a common issue in rodent models [24]. For a less virulent organism, investigations into a more pathogen-specific probe would be beneficial for diagnosing and monitoring these infections.

The first 2 scans for *S. epidermidis*-infected animals occurred earlier than for *S. aureus*. A direct SUV_max_ comparison at those points is not possible, but the final scan is overall comparable.

## CONCLUSION

FDG-PET could diagnose rats with an untreated *S. aureus* VGEI. However, when comparing rats with a suppressed infection to uninfected rats, FDG uptake was similar. Histological analysis revealed comparable decreases in inflammatory responses between the 2, providing a possible explanation for the observation, especially in the context of pathogen-induced increases in IL-10 levels. This implies that potentially suppressed infections may incorrectly appear to be cured in FDG-based imaging modalities, highlighting the need for pathogen-specific imaging methods. Given the severity of VGEI and the difficulties associated with monitoring treatment response, this study adds valuable insights into the kinetics of FDG uptake in relation to both bacterial burden and inflammation in the host.

## Supplementary Material

jiaf594_Supplementary_Data

## Supplementary Data

Supplementary materials are available at *The Journal of Infectious Diseases* online (http://jid.oxfordjournals.org/). Supplementary materials consist of data provided by the author that are published to benefit the reader. The posted materials are not copyedited. The contents of all supplementary data are the sole responsibility of the authors. Questions or messages regarding errors should be addressed to the author.

## Notes

***Acknowledgments.*** We want to thank Mette Simonsen (Department of Nuclear Medicine, Aarhus University Hospital) for her help with PET scans. We want to thank Lone Pødenphant (Department of Clinical Microbiology, Aarhus University Hospital) for helping with supplying and delivering agar plates.

***Financial support.*** This work was supported by a PhD research grant from the Department of Clinical Medicine, Aarhus University (EF) and Knud og Edith Eriksens Mindefond.

Funding sources were not involved in the study design, data collection, analysis, interpretation, report writing, or the decision to submit the article for publication.

***Meetings where the information has previously been presented.*** Parts of the study were presented at an oral presentation at ISCVID (International Society for Cardiovascular Infectious Diseases) 2024, Malmö, Sweden, under the title “Infection or inflammation – what is the primary driver for PET/MRI positive FDG uptake in a *Staphylococcus aureus* vascular graft infection rat model?”.

***Author Contributions.*** E. F., R. L. M., L. Ø., M. H. V., and N. P. J. contributed to the study design. E. F., M. I. J., C. G., C. B. R., and L. K. J. contributed to data collection. E. F., C. B. R., L. K. J., M. H. V., and N. P. J. contributed to data analysis. E. F., C. B. R., L. K. J., M. H. V., and N. P. J. contributed to writing the original draft. All authors contributed to review and editing of the manuscript.

***Declaration of Generative AI and AI-assisted technologies in the writing process***. While preparing this work, the authors used Grammarly to improve clarity and language. After using this tool, the authors reviewed and edited the content as needed and take full responsibility for the publication's content.
