# Supplementary material for: [18F]Fluorodeoxyglucose Positron Emission Tomography for Diagnosis and Monitoring of Acute Staphylococcus aureus Vascular Graft Infection in a Rat Model
Source: J Infect Dis. 2025 Nov 26;233(2):e332–41. doi: 10.1093/infdis/jiaf594 (PMC13017435; doi:10.1093/infdis/jiaf594)
Supplement: jiaf594_Supplementary_Data [file jiaf594_supplementary_data.zip › Supplementary_FigS1.docx]

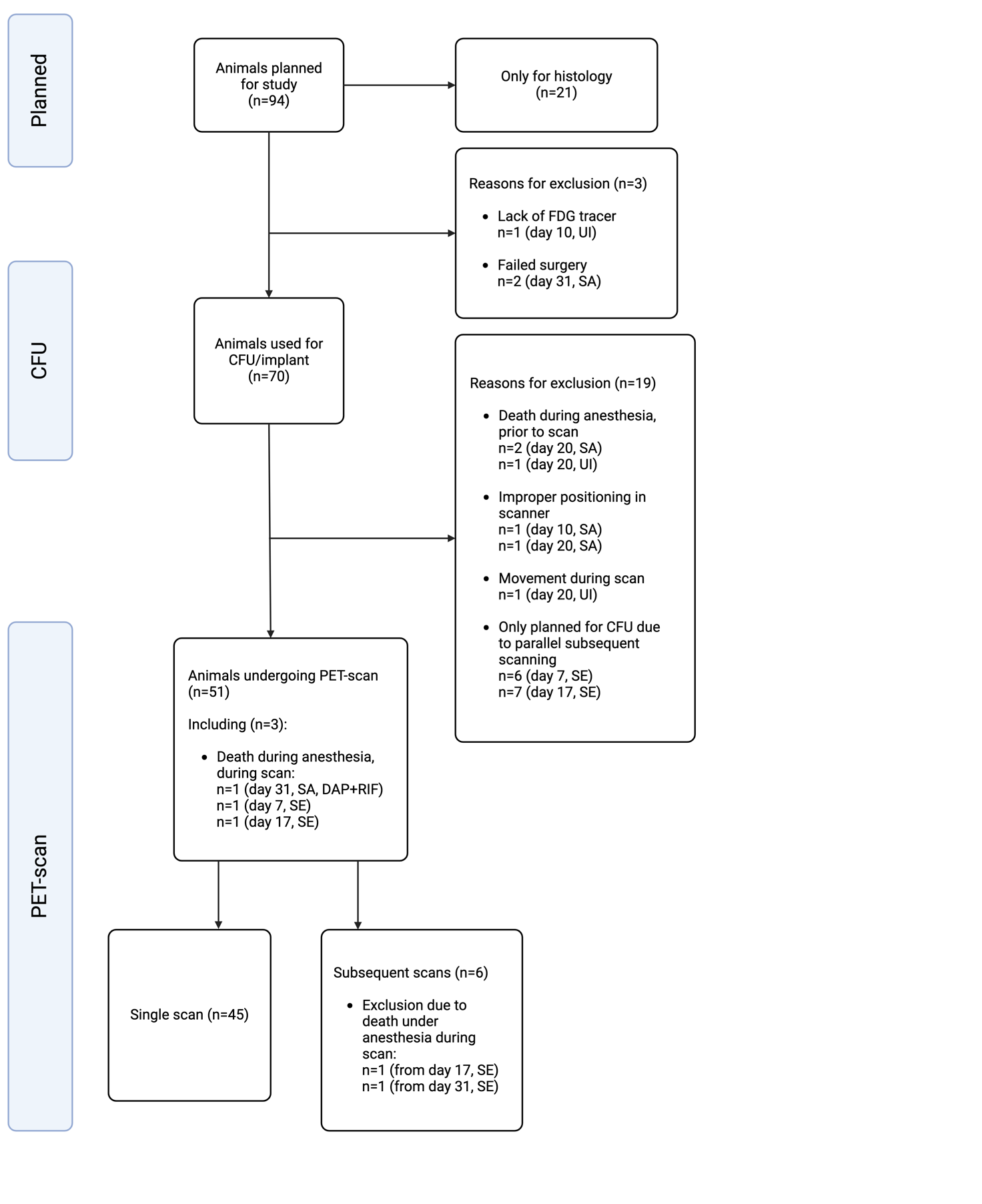


**Supplementary figure S1.** Animal deaths and exclusion from study. SA = *S. aureus*. SE = *S. epidermidis*. UI = uninfected. Created in BioRender. Faddy, E. (2025) https://BioRender.com/b76c419
