# Supplementary material for: [18F]Fluorodeoxyglucose Positron Emission Tomography for Diagnosis and Monitoring of Acute Staphylococcus aureus Vascular Graft Infection in a Rat Model
Source: J Infect Dis. 2025 Nov 26;233(2):e332–41. doi: 10.1093/infdis/jiaf594 (PMC13017435; doi:10.1093/infdis/jiaf594)
Supplement: jiaf594_Supplementary_Data [file jiaf594_supplementary_data.zip › Supplementary_FigS2.docx]

**
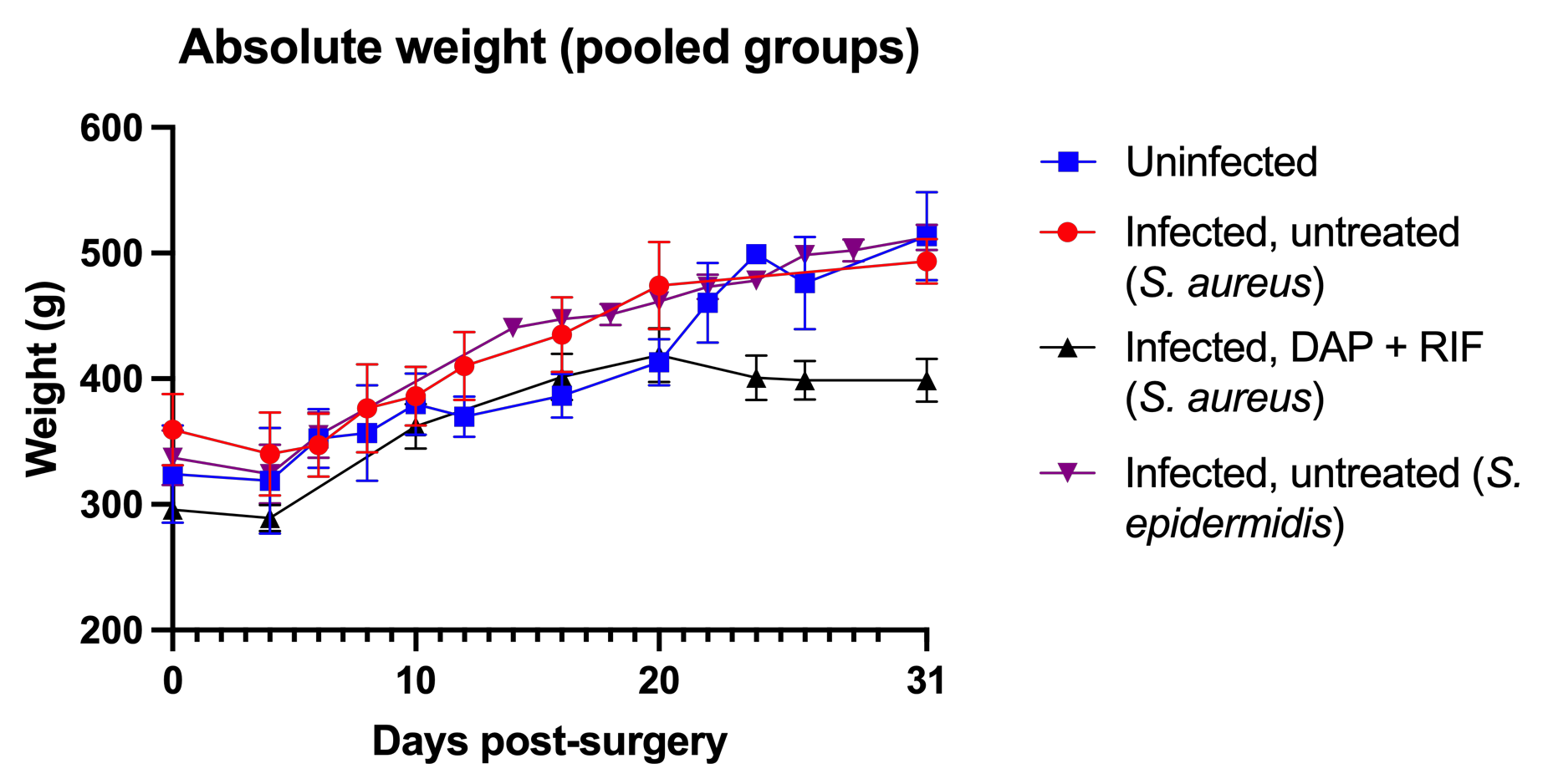
**

**Supplementary figure S2.** Absolute weight of rats. Each point represents mean of pooled group, bars represent SD.
