# Supplementary material for: [18F]Fluorodeoxyglucose Positron Emission Tomography for Diagnosis and Monitoring of Acute Staphylococcus aureus Vascular Graft Infection in a Rat Model
Source: J Infect Dis. 2025 Nov 26;233(2):e332–41. doi: 10.1093/infdis/jiaf594 (PMC13017435; doi:10.1093/infdis/jiaf594)
Supplement: jiaf594_Supplementary_Data [file jiaf594_supplementary_data.zip › Supplementary_FigS3.docx]

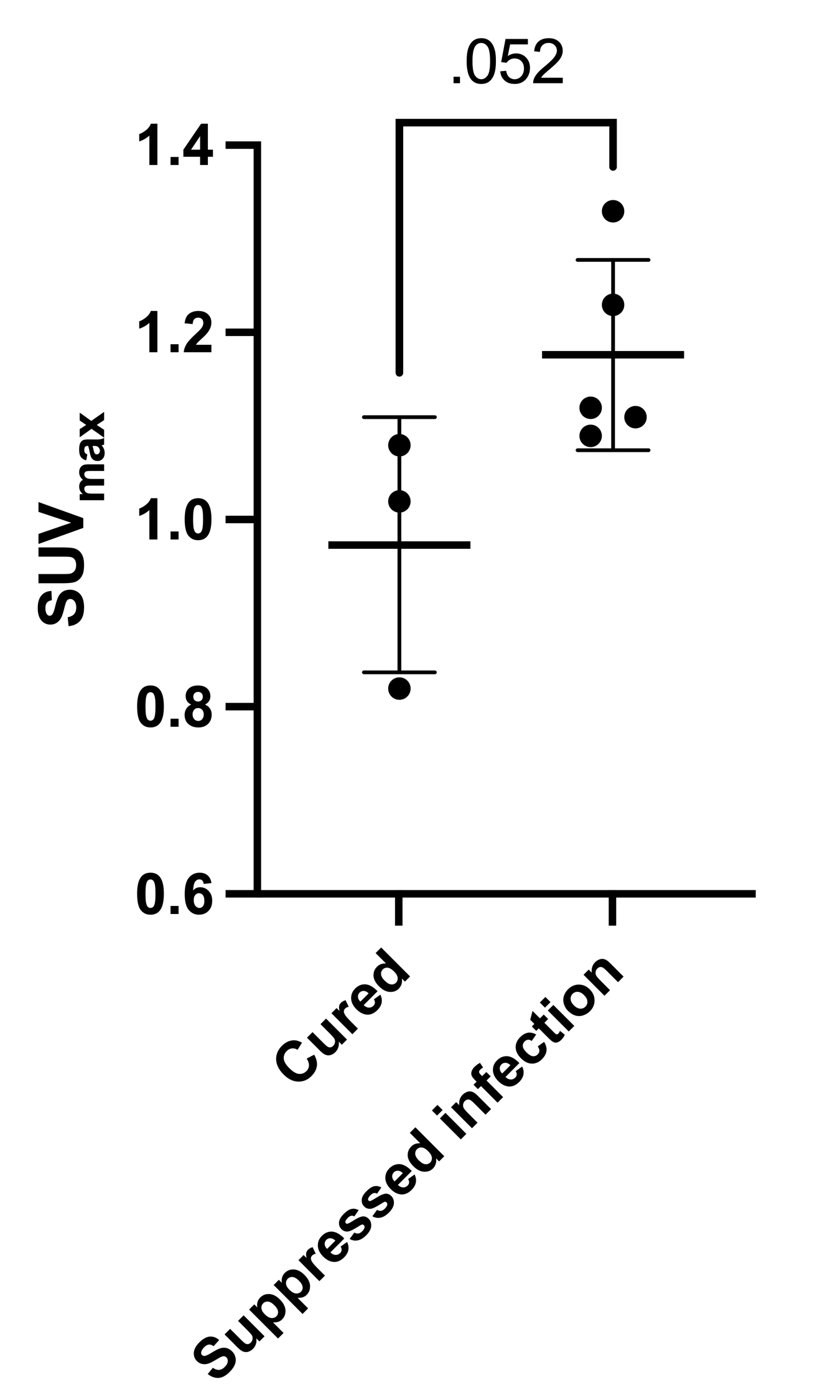


**Supplementary figure S3.** SUV_max_ values for rats scanned at day 31 infected with *S. aureus* and treated with daptomycin and rifampicin. Rats are divided into two sub-groups based on CFU values *ex vivo*. n = 3 cured (CFU = 0). n = 5 suppressed infection (CFU > 0). Each data point represents SUV_max_ from one rat. Bars represent mean and SD. Unpaired t-test.
