# Supplementary material for: [18F]Fluorodeoxyglucose Positron Emission Tomography for Diagnosis and Monitoring of Acute Staphylococcus aureus Vascular Graft Infection in a Rat Model
Source: J Infect Dis. 2025 Nov 26;233(2):e332–41. doi: 10.1093/infdis/jiaf594 (PMC13017435; doi:10.1093/infdis/jiaf594)
Supplement: jiaf594_Supplementary_Data [file jiaf594_supplementary_data.zip › Supplementary_Text.docx]

**Supplementary Text for**

**[^18^F]Fluorodeoxyglucose Positron Emission Tomography for diagnosis and monitoring of acute *Staphylococcus aureus* vascular graft infection in a rat model**

**Authors:** Emma Faddy^a,b,c **^, Mikkel Illemann Johansen^b^, Christoffer Gadeberg^a,b^, Rikke Louise Meyer^d,e^, Lars Østergaard^a,b^, Cecilie Bay-Richter^f^, Louise Kruse Jensen^g^, Mikkel Holm Vendelbo^h,i^, Nis Pedersen Jørgensen^b,j *^

**Affiliations:**

^a^ Department of Clinical Medicine, Infectious Diseases, Aarhus University, Palle Juul-Jensens Blvd. 99, Aarhus N, Denmark.

^b^ Department of Infectious Diseases, Aarhus University Hospital, Palle Juul-Jensens Blvd. 99, Aarhus N, Denmark.

^c^ Department of Orthopedic Surgery, Regional Hospital of Randers, Skovlyvej 1, 8930 Randers NØ, Denmark.

^d^ Interdisciplinary Nanoscience Center (iNANO), Aarhus University, Gustav Wieds Vej 14, Aarhus C, Denmark.

^e^ Department of Biology, Aarhus University, Ny Munkegade 114, Aarhus C, Denmark.

^f^ Translational Neuropsychiatry Unit, Department of Clinical Medicine, Aarhus University, Denmark.

^g^ Department of Veterinary- and Animal Sciences, University of Copenhagen, Frederiksberg C, Denmark.

^h^ Department of Nuclear Medicine & PET Centre, Aarhus University Hospital, Palle Juul-Jensens Boulevard 165, 8200 Aarhus N, Denmark.

^i^ Department of Biomedicine, Aarhus University, Høegh-Guldbergs Gade 10, 8000 Aarhus C, Denmark

^j^ Department of Orthopedic Surgery, Aarhus University Hospital, Palle Juul-Jensens Boulevard 165, 8200 Aarhus N, Denmark.

*** Corresponding author:** Nis Pedersen Jørgensen. Email: [nisjoerg@rm.dk](mailto:nisjoerg@rm.dk), Phone: +45 25 37 20 55

**** Alternate contact author:** Emma Faddy. Email: [emmfad@rm.dk](mailto:emmfad@rm.dk), Phone: +45 60 77 88 16

**Animal deaths and exclusion from study.**

During preterminal scans, three rats (n = 2 day 20, *S. aureus*-infected, untreated; n = 1 day 20, uninfected) died during anaesthesia, most likely due to asphyxia (Fig. S1). CFU measurements from implants were still possible to obtain from these rats and were included in Fig. 4. Scans for these rats were not available.

Three rats were alive when entering the scanner but had died at the end of the scan during anaesthesia (n = 1 day 31, *S. aureus*-infected, DAP+RIF; n = 1 day 7, *S. epidermidis*-infected; n = 1 day 17, *S. epidermidis*-infected). As FDG would have been distributed in the tissue and bound prior to death, these scans were included.

Due to the size of the rats, PET scans were conducted without adjunctive anatomical imaging modality. Consequently, two rats (n = 1 on day 10, S. aureus infected, untreated; n = 1 on day 20, S. aureus infected, untreated) were improperly positioned within the scanner. This misalignment led to the region of interest being outside the imaging frame. Due to movement, one rat (n = 1 day 20, uninfected) could not be analysed.

One rat (n = 1 day 10, uninfected) was not scanned due to a shortage of FDG tracer on scanning day.

Two rats (n = 2 day 31, *S. aureus*-infected, untreated) were excluded from the study, as problems with implantation in these specific rats had been noted during surgery, resulting in uninfected implants by day 31.

**ARRIVE.**

Randomisation: It was not possible to randomise group allocation of rats due to logistics surrounding dedicated slots for PET/MRI scans. The scan order of the rats was randomised each scanning day by letter randomisation. The operator of the PET/MRI scanner was blinded to the groups, except for the *S. epidermidis-*infected rats, which were housed at the PET/MRI facilities, in contrast to the other groups.

**Weight of animals during the study.**

In all groups, an initial drop in weight was seen in the first four days after surgery (Fig. S2). By day 8, the weight had returned to baseline, and, except for the treatment group, rats in all groups continued to gain weight throughout the experiment. The group infected with *S. aureus* undergoing treatment with daptomycin and rifampicin had a small decrease in weight (mean ±SD) from 418.9 g ± 21.36 g at day 20 to 400.8 g ± 7.81 g by day 24 (p < .0001, paired t-test), corresponding to a decrease in weight of 4,3%, which coincided with the start of treatment at day 20. The weight stabilised by day 26 (398.8 g ± 15.47 g) but did not increase further for the duration of the study. The weight decrease due to this treatment has previously been described and is a well-known side effect of the antibiotics^15^.
